# Increased Resin Collection after Parasite Challenge: A Case of Self-Medication in Honey Bees?

**DOI:** 10.1371/journal.pone.0034601

**Published:** 2012-03-29

**Authors:** Michael D. Simone-Finstrom, Marla Spivak

**Affiliations:** 1 Department of Entomology, North Carolina State University, Raleigh, North Carolina, United States of America; 2 Department of Entomology, University of Minnesota, St. Paul, Minnesota, United States of America; AgroParisTech, France

## Abstract

The constant pressure posed by parasites has caused species throughout the animal kingdom to evolve suites of mechanisms to resist infection. Individual barriers and physiological defenses are considered the main barriers against parasites in invertebrate species. However, behavioral traits and other non-immunological defenses can also effectively reduce parasite transmission and infection intensity. In social insects, behaviors that reduce colony-level parasite loads are termed “social immunity.” One example of a behavioral defense is resin collection. Honey bees forage for plant-produced resins and incorporate them into their nest architecture. This use of resins can reduce chronic elevation of an individual bee's immune response. Since high activation of individual immunity can impose colony-level fitness costs, collection of resins may benefit both the individual and colony fitness. However the use of resins as a more direct defense against pathogens is unclear. Here we present evidence that honey bee colonies may self-medicate with plant resins in response to a fungal infection. Self-medication is generally defined as an individual responding to infection by ingesting or harvesting non-nutritive compounds or plant materials. Our results show that colonies increase resin foraging rates after a challenge with a fungal parasite (*Ascophaera apis*: chalkbrood or CB). Additionally, colonies experimentally enriched with resin had decreased infection intensities of this fungal parasite. If considered self-medication, this is a particularly unique example because it operates at the colony level. Most instances of self-medication involve pharmacophagy, whereby individuals change their diet in response to direct infection with a parasite. In this case with honey bees, resins are not ingested but used within the hive by adult bees exposed to fungal spores. Thus the colony, as the unit of selection, may be responding to infection through self-medication by increasing the number of individuals that forage for resin.

## Introduction

Organisms have evolved a multitude of defenses to resist or tolerate parasitic infection [1], [2]. Parasites can include macroparasites such as arthropods and microparasites such as bacteria and fungi that live on or in a host and reduce overall fitness. Individual barriers and physiological mechanisms, such as a cuticle and inducible antimicrobial peptides in insects, are common modes of coping with infection; however organisms also exhibit various behavioral traits to contend with parasites [2], [3]. Social species add another layer of complexity in their defense repertoires because the defenses function at both the individual and group levels. In eusocial insects (e.g. honey bees and ants), physiological and behavioral defenses are observable and have fitness consequences for both the individuals and colony. Social immunity is the phenomenon in which the behavior of the individual reduces the parasite load and parasitic stress at the group level [4]. For example, the incorporation of plant resins in a honey bee (*Apis mellifera*) nest interior has been shown to reduce colony bacterial loads and reduces overall investment in individual immune function, which may positively affect colony fitness [5], [6]. The research presented here provides evidence that the use of resins by honey bees may be an example of a colony-level mechanism of self-medication, supporting the concept that resin collection in honey bees is a form of social immunity. To truly classify a trait as self-medication in animals, it should be adaptively plastic, meaning that an individual (or colony in this case) should perform the behavior at higher rates when parasitized and at lower rates or not at all when healthy [7].

The best-studied examples of self-medication include ingestion of whole leaves by individuals of various primate species to eliminate nematode infections [8]–[11], or the ingestion of secondary plant metabolites most notably by various caterpillars and bumble bees [7], [12]–[14]. These examples describe behaviors that fall under the term pharmacophagy, which is the ingestion of non-nutritive substances for purposes other than energetic demands [15].

Other types of self-medication involve the use of whole leaves and plant secondary metabolites externally, rather than through ingestion. Pharmacophory defines these behaviors in which plant materials are collected and used externally [16] (e.g. in nest construction or grooming behaviors [5], [6], [17]–[21]). In either cases of pharmacophory or pharmacophagy, the behavior may be constitutively expressed and thus prophylactic, rather than conditionally expressed and thus a form of self-medication. For example, several studies have determined that aromatic leaves used in nest construction by European starlings and blue tits may negatively offset parasite load and positively affect fledgling immunocompetence [18], [21]. These birds do not appear to increase collection in response to high parasite loads therefore it is likely a prophylactic behavior. Another example of resin use was illustrated in a series of laboratory-based studies with the social Swiss wood ant (*Formica paralugubris*). Resin use within a nest is a form of social immunity because it reduces overall microbial load within the colony [19] and can lead to increased survival of parasitized individuals [22], [23]. While this behavior is a fascinating example of pharmacophory—the ants use resin prophylactically to benefit the colony—it was found not to be an example of self-medication, as individuals do not increase resin collection when parasite-challenged [24].

Honey bees collect resins from a variety of plant species worldwide. In temperate regions, it is commonly thought that *Populus* spp. are the main sources, while in tropical regions resin-producing floral resources (e.g., *Clusia* spp.) and herbaceous shrubs (e.g., *Baccharis dracunculifolia*) are commonly used [6], [25]–[27]. “Propolis” (Greek for “pro”—in front or defense of—“polis”—the city) is the apicultural term for honey bee-collected resins used within a hive. Non-managed, feral honey bee colonies, typically living in tree cavities, line the entire hive interior with a thin layer of resin mixed with varying amounts of wax in what has been termed the “propolis envelope” [6], [28].

Our previous studies have shown that honey bees in a resin-enriched hive are able to reduce individual investment in immune function due to an overall decrease in colony bacterial loads [5]. Similar to its function in Swiss wood ant nests [19], resin use by honey bees is prophylactic pharmacophory and functions as a type of social immunity [4], whereby the incorporation of resins in the nest by individual honey bees benefits colony-level immunity.

Here we go a step further, asking if honey bee colonies self-medicate by collecting plant-produced resins in addition to using them prophylactically. We monitored foraging rates of colonies before and after colony-level exposure to microbial parasites. This study was conducted from early July to early September in 2008, 2009, and 2010. In all three years, colonies were exposed to the fungal parasite *Ascophaera apis*, the causative agent of the larval disease chalkbrood (CB). In 2009, additional colonies were challenged with the bacterial parasite *Paneabacillus larvae* (the causative agent of the larval disease American foulbrood, or AFB) or were exposed to spores of the soil-borne fungal entomopathogen *Metarhizium anisopliae*. Since *Metarhizium* is not pathenogenic to honey bees, it was used as a control for increased microbial loads in challenged colonies.

## Results

**Figure 1:**
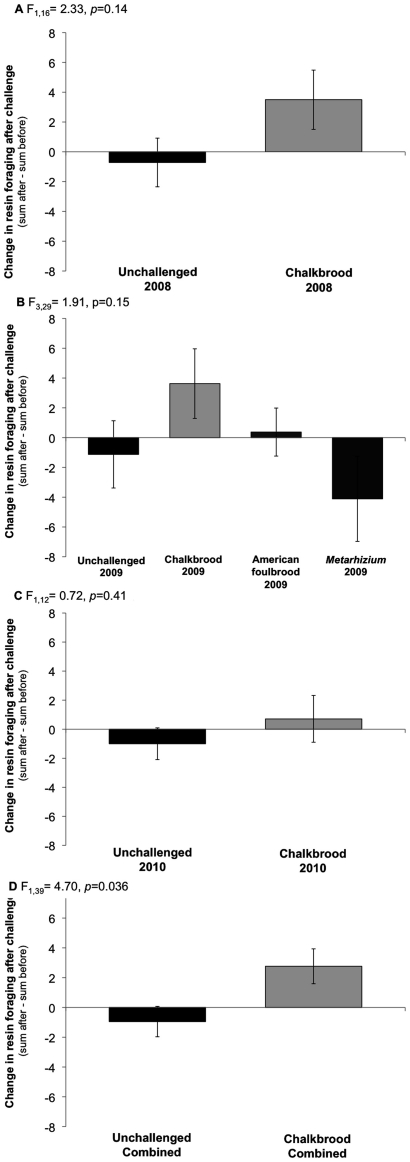
Change in resin foraging rates before and after challenge. Data were analyzed using ANOVA for each series with p-values reported within each graph. A) 2008 (n = 7 unchallenged, n = 10 challenged); B) 2009 (n = 8 for unchallenged, chalkbrood, and American foulbrood; n = 9 for *Metarhizium*; C) 2010 (n = 6 unchallenged, n = 7 challenged); D) Data combined for unchallenged and chalkbrood challenge treatments over the three years of study.

Overall, challenge with CB increased resin collection by honey bee colonies (Fig. 1A–D). All means are reported with their standard error. Across the three years for the CB-challenged colonies, the mean number of resin foragers before challenge was 6.0±1.0, while the mean after challenge was 8.7±0.9 per 15 min observation period. In unchallenged colonies, there was a mean of 9.1±1.4 resin foragers pre-challenge and 8.2±1.5 post-challenge. The mean difference in the total number of resin foragers, (sum after – sum before challenge), was 2.8±1.1 for CB-challenged colonies and −0.9±1.2. Overall resin foragers consistently represented a minority of the foraging force (∼1% or less of total number of foragers), so this seemingly small change in the mean number of bees foraging for resins within a 15 min interval is biologically significant.

A two-way ANOVA of the combined data modeling CB-treatment and year as main effects determined that there was a significant increase in resin foraging rates due to CB-challenge (F_1,46_ = 4.70, *p* = 0.036; Fig. 1D). There was no effect of year (F_2,46_ = 0.33, *p* = 0.72) or interaction between year and CB-challenge (F_2,46_ = 0.30, *p* = 0.74). Analysis of the years independently indicated that in 2008 there was a moderate, but non-significant increase in resin-foraging after CB-challenge (F_1,16_ = 2.33, *p* = 0.14; Fig. 1A). In 2009 colonies were challenged with two other pathogens. Whole model analysis indicated that resin foraging in CB-challenged colonies increased significantly relative to *Metarhizium*-challenged colonies (*p* = 0.025), and moderately relative to unchallenged colonies (*p* = 0.17). The change in total number of resin foragers seen for AFB-challenged colonies was not significantly different from any treatment (*p*>0.18; refer to Fig. 1B). In 2010 there was a non-significant increase in resin foraging rates after CB challenge, (F_1,12_ = 0.72, *p* = 0.41; Fig. 1C).

**Figure 2:**
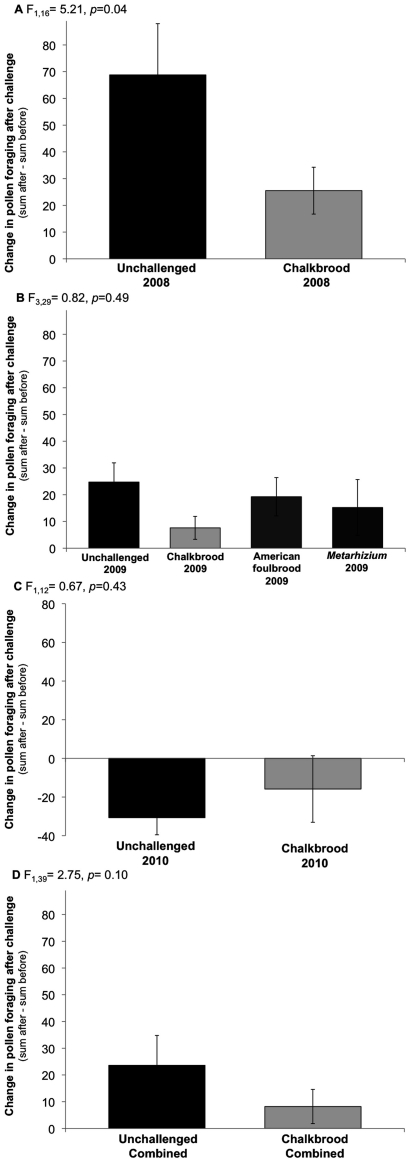
Change in pollen foraging rates before and after challenge. Data were analyzed using ANOVA for each series with p-values reported within each graph. A) 2008 (n = 7 unchallenged, n = 10 challenged); B) 2009 (n = 8 for unchallenged, chalkbrood, and American foulbrood; n = 9 for *Metarhizium*); C) 2010 (n = 6 unchallenged, n = 7 challenged); D) Data combined for unchallenged and chalkbrood challenge treatments over the three years of study.

The increase in the rate of resin foraging post-challenge was not a result of an increase in general foraging rates in CB-challenged colonies, as indicated by the pollen forager counts (Fig. 2A–D). Pollen foragers were very abundant, as expected, especially with respect to resin foragers. On average over the three years, CB-challenged colonies had 50.3±5.3 pollen foragers pre-challenge and 58.5±4.7 post-challenge per 3 min (c.f., per 15 min for resin foragers). In contrast, the means (+SE) for unchallenged colonies were 54.6±6.5 pre-challenge and 78.2±8.0 post-challenge. This resulted in mean overall differences (sum after – sum before) in total pollen foragers between the pre- and post-challenge periods of 8.2±6.2 for the CB-challenged colonies and 23.6±6.7 for the unchallenged colonies. The two-way ANOVA modeling treatment and year as main effects for the combined data determined that CB-treatment did not significantly influence the change in pollen foraging pre- and post-challenge (F_1,46_ = 2.75, *p* = 0.10; Fig. 2.D). An effect of year was determined with the largest increase in pollen collection irrespective of CB-treatment in 2008, followed by 2009, and in 2010 there was a slight decrease (F_2,46_ = 19.02, *p*<0.0001). Analysis of the individual years indicated that in 2008 unchallenged colonies had a significantly higher increase in pollen foraging post-challenge compared to CB-challenged colonies (F_1,16_ = 5.21, *p* = 0.04; Fig. 2A). There was no significant difference due to treatment in 2009 (F_3,29_ = 0.82, *p* = 0.49; Fig. 2B) or in 2010 (F_1,12_ = 0.67, *p* = 0.43; Fig. 2C).

In addition to the increase in resin foraging after CB challenge, we found that resins may play a role as a direct defense against this fungal parasite in a honey bee colony. Half of the colonies in 2008 were made resin-rich by painting interior hive walls with propolis extracts using previously established methods [5]. Three weeks after challenge, the CB-challenged, resin-poor colonies had a significantly higher total infection compared to CB- challenged, resin-rich and unchallenged colonies (resin treatment and CB-challenge interaction effect: F_1, 16_ = 4.78, p = 0.04; Table 1).

**Table 1:** Chalkbrood infection levels in colonies in 2008.

Colony	Challenge	CB Mummies	CB Mummies	Total	
Treatment		Count 1	Count 2		
Resin-poor	Unchallenged	0	0	0	**A**
	Chalkbrood	42.3±25.1	65.8±38.2	108.2±49.0	**B**
Resin-rich	Unchallenged	3.2±1.6	2.2±1.4	5.3±1.7	**A**
	Chalkbrood	1±0.5	13.7±7.2	14.7±7.5	**B**

The data is from all colonies used in 2008 regardless of use in analyses of foraging rates (n = 5 resin-poor unchallenged colonies; n = 6 each for resin-poor challenged, resin-rich unchallenged and resin-rich challenged colonies). Resin-rich unchallenged colonies positive for CB-infection either had persistent low levels of infection (1 colony) or low-levels of infection at only one time point (2 colonies). Letters indicate significant differences in the total number of mummies based on two-way ANOVA.

## Discussion

We have shown that the rate of resin foraging increased when free-flying honey bee colonies were exposed to the fungal agent of the larval disease CB (*A. apis*), suggesting that honey bee colonies may be self-medicating in response to this particular pathogen. The data presented here also suggests that in addition to indirect immune effects, resins may play a direct role against a specific fungal parasite. While there is evidence that propolis extracts are effective *in vitro* against a variety of fungal parasites, limited previous knowledge on the activity of propolis against CB exists [6]. The results presented here as part of the 2008 study suggest that a resin-rich environment may directly reduce CB-infection intensity and may have an inhibitory effect on the growth of this fungus. In this regard, resin collection by honey bee colonies could be a novel instance of self-medication where individual expression of a behavioral trait is altered due to exposure to a fungal pathogen. Honey bees do not ingest resin, and CB parasitizes larvae—not adults—thus the collection of resin by adults affects colony health, or social immunity. If honey bee foragers are in fact responding to certain parasites by increasing the collection of antimicrobial resins, this is as a particularly unique example of self-medication. Since in highly eusocial insects (e.g. honey bees, ants, termites) the colony is the reproductive unit and the focus of selection [29], [30], the colony can be viewed as the “self” in this sense.

Resin foraging is relatively rare, particularly in European-derived bees. The domestication of honey bees has resulted in a reduction of resin collection [31], likely because beekeepers have selected against its use since the presence of large quantities of sticky propolis often makes opening hives more difficult. The amount of propolis lining a natural nest cavity, such as the propolis envelope in a tree cavity [6], has not been quantified, but we found that creating a propolis envelope containing 60 g of resin positively affected colony-level immunity [5]. Thus an increase in the number of resin foragers, even a relatively slight increase as seen in the present study, likely has a large biological effect. While we detected a statistically significant effect of CB-challenge on resin foraging rates after combining the three years of study, the trends in each year suggest that increasing the power in a single year would produce the same result. In 2010, it is possible that foraging rates were low overall, as indicated by the general decrease in pollen foraging post-challenge, so even the modest increase in resin foraging that we found suggests the importance that this behavior may have at the colony-level.

Most other instances of self-medication seen in vertebrates and invertebrates involve individuals changing their diet (pharmacophagy) in response to direct infection with a parasite [7], [10]. In this case with honey bees, individuals are using the resins within the nest (pharmacophory) and not ingesting them [16]. Furthermore the colony is responding to parasitic infection, not an individual per se. The following highlight the difference between our research with honey bees and the other clear examples of self-medication: chimpanzees with active nematode infections swallow whole leaves [8], [9], [32]; parasitoid-infected *G. incorrupta* caterpillars ingest non-nutritive alkaloids [7]; *Spodoptera littoralis* caterpillars preferentially consume high protein diets when infected with a virus [12]. In the latter two examples with solitary insects, the ingestion of these compounds results in strong fitness costs (e.g. shortened lifespan) when individuals are not infected. Although bees do not consume resins, foraging for resin is likely costly at the individual level because it is time-consuming to handle resin both at its source and in the hive, and provides no obvious direct food reward as does foraging for nectar or pollen. However, resin collection does function as a mechanism of social immunity [6]. The incorporation of resin in the nest environment reduces general bacterial loads in the colony, either by inhibition due to direct contact or by the volatile compounds released [33], and therefore allows individuals to invest less in immune function [5]. Since high activation of individual immunity can have colony-level fitness costs [34], traits that reduce chronic elevation of an individual's immune response may be of benefit to colony productivity. Therefore any costs to the individual may be offset by the benefits of resin collection to the colony, since individual fitness is largely determined by colony success in honey bees.

Since only larvae can become infected with CB, it was at first surprising that adults altered their behavior in response to increased levels of a parasite that does not directly affect them. However, from a social immunity perspective, the colony is the infected unit and so the colony responded by increasing the number of resin foragers. Our finding that the level of resin collection only changed after challenge with CB and not AFB or *Metarhizium* warrants further study. To challenge with CB, we homogenized CB spores within a mixture of pollen, thus adult bees handled and possibly ingested them. Spores do not germinate within the gut of an adult bee but can remain there, and adults are the major distributor of CB spores throughout a colony through larval feeding [35]–[37]. Individual adult bees were therefore exposed to an overall increase in fungal spores throughout the colony as a result of the CB challenge, even though colonies largely exhibited mild disease symptoms or lacked clinical symptoms. A lack of a similar response in the colonies treated with the entomopathogen *Metarhizium* could be due to the fact that honey bees are not normally exposed to this type of soil-borne fungus. Honey bees routinely remove debris from the floor of the colony [38], which was largely where the *Metarhizium* was within the challenged colonies. Since this fungus did not replicate and accumulate in the hive, it is likely that *Metarhizium*-challenged colonies focused on simply removing the powder. However, the studies done on resin use *Formica paralugubris* after challenge with *Metarhizium*, which does naturally infect this species, also found that these ants do not increase collection after exposure to this parasite [24] even though the presence of resin can help reduce mortality. So it is possible this type of self-medication is a more specific response in honey bees and perhaps other species, although more research needs to be conducted on this front.

It is not clear why colonies did not increase resin collection in response to AFB bacterial challenge. While cellular immune mechanisms (e.g., cellular encapsulation) are likely involved in the individual defense against fungal parasites [39], [40], the suite of physiological defenses, particularly the antimicrobial peptides, of honey bees appear to be geared more toward controlling bacterial parasites [41]. However, propolis extracts have been shown to exhibit activity against AFB in laboratory cultures [42] and in field colonies fed propolis extracts in sugar syrup [43]. A resin-rich environment also has been shown to reduce the general bacterial loads in colonies [5]. Thus it would seem that self-medicating with resin against bacterial infection, in addition to a fungal infection, would be an adaptive response, and warrants further study. Another aspect that may influence a response to parasites with some defense mechanisms and not others is simply that there are a number of defenses that individuals and colonies can often use against a single parasite (e.g. hygienic behavior, resin collection, grooming, fever response, physiological measures [2]). Given this, it is currently unknown how these suite of defenses are used within a single colony and how they are used in concert.

In the study presented here and the work done with the caterpillar *G. incorrupta* [7], parasitism increases the rate at which a routine behavior is performed instead of the initiation of an atypical behavior (e.g., leaf-swallowing in primates [8]–[11]). The behavioral mechanism involved in the initiation of honey bee resin foraging in response to colony-level challenge with a specific fungal parasite is currently unknown. In cases of self-medication in vertebrates, associative or social learning is typically involved [11], [44], [45]. However, the few insect cases that exhibit self-medication do not necessarily involve learning but rather appear to be responses to alteration in the organism's homeostasis. In *G. incorrupta*, infection causes changes in an individual's peripheral nervous system and heightens activity of taste receptors for pyrrolizidine alkaloids (PA), leading to increased consumption of PA-rich food sources [46]. One possible cue that resin foragers may use to initiate resin foraging in response to CB levels could be olfactory stimuli. Larvae release specific chemical compounds in response to CB infection and these compounds induce hygienic behavior, a type of social immunity in which bees remove diseased larvae and pupae from the nest [47]. These compounds are released prior to clear visual development of clinical symptoms [47]. Resin foragers may also use cues related more directly to colony microbe levels. Since feral colonies line the entirety of the nest interior with resins prior to and during comb construction, it is possible that bacteria and fungi normally found in a tree cavity may also induce the behavior. There are likely many other stimuli involved in the behavior that are non-mutually exclusive [48]. For example, since this is self-medication at the colony-level, social stimuli must also be considered, and resin foragers have been noted to dance as a possible mechanism of forager recruitment [26], [49].

A host of questions still exist concerning resin collection and use by honey bees, as well as resin use across the animal kingdom in general. Its role as a mechanism of social immunity in bees and ants is likely quite complex, involving direct effects against parasites and more indirect effects on individual immunity. The sequestering of resins and secondary plant metabolites appears to be a relatively widespread trait, and many species may utilize these plant defenses as a mechanism of defense against various parasites and predators. While we have some evidence that resin collection by honey bees may be a novel case of pharmacophorous self-medication in an insect, it is possible that this phenomenon is more widespread than previously thought.

## Materials and Methods

### Colony setup

Colonies in 2008 were matched for population size (∼8,000 adult bees) and maintained in new, single standard Langstroth beekeeping boxes containing 9 frames of comb. Throughout the course of the experiment, colonies were maintained with one brood box. Colonies were divided between two apiaries in southeastern Minnesota. Twelve colonies were made resin-rich by painting the inside walls with approximately 92.5 g of MN-derived propolis extract in 70% EtOH to mimic the propolis envelope seen in feral honey bee colonies [5]. Eleven colonies were left resin-poor and painted with the same volume of 70% EtOH.

In 2009 and 2010, colonies were established in “nucleus” boxes with four frames of comb and equal numbers of adult bees and naturally-mated sister queens. All colonies were maintained in the same apiary.

### Parasite Challenge

Colonies were challenged with the fungal pathogen, *Ascosphaera apis*, by homogenizing fresh chalkbrood (CB) mummies (i.e., dead bee larvae infiltrated with mycelia) and mixing into pollen substitute and 50% sucrose solution (2008) or into mixed-source pollen and 50% sucrose solution (2009, 2010) modified from Gilliam et al. [35]. In 2008, 12 colonies were given approximately 3.3 mummy equivalents in 450 g pollen patties. In 2009 and 2010, 9 and 7 colonies, respectively, were given 10 mummy equivalents in 75 g pollen patties. Control colonies were given pollen patties without CB. The level of CB infection was determined by counting the number of mummies present in comb cells within each colony at the midpoint and at the end of the experimental period. In 2009, an additional nine colonies were used for each of the three following parasite-challenge treatments, and nine colonies were used as unchallenged controls. For the AFB-challenge a 7.5 cm square section of comb from a colony infested with American foulbrood (AFB, *Paenibacillus larvae*) that contained AFB larval scales was introduced into 9 colonies [50]. For exposure to *Metarhizium anisopliae* (an entomopathogenic fungus that does not to infect honey bees) the inside floors of the remaining 9 colonies were dusted with 75 g of *M. anisopliae* ECS1 powder containing approximately 1×10^10^ conidia per gram. This amount has been shown to not adversely affect colony development or health [51], and was used as a control for an increase in microbial loads in challenged colonies. All treatments were completed twice during a two-week challenge period. Levels of infection due to the challenges were measured once in the midpoint and at the end of the experiment.

### Resin collection

The number of resin and pollen foragers returning to the hive during the pre-challenge (July) and post-challenge (August) period was determined by closing the colony entrance between 1200h and 1600h each day for four (2008) or six (2009, 2010) days over two weeks. The number of bees with resin (15 min after colony closure) or pollen (3 min after) on their corbiculae were counted without replacement. Since resin foragers are relatively rare (∼1% of all foragers), a 15-minute observation period was deemed sufficient to collect adequate numbers of resin foragers while preventing closed colonies from overheating. Pollen foragers were used as a proxy for total foraging force as they have clearly been foraging; other bees flying to and from the hive could be guard bees or on orientation flights and not true foragers. Since resin collection can vary throughout a season, data collection was limited to two-weeks pre-challenge and two-weeks post-challenge. By monitoring the number of resin foragers that return to the colony over a given period, we were able to accurately measure the change in resin collection over the pre- and post-challenge periods. Other measures of resin collection (e.g. in-hive deposition) are inaccurate since resin is placed throughout the nest interior in small cracks and crevices and mixed with varying amounts of wax.

### Data analysis

To describe change in resin and pollen foraging across the two time periods in the most straightforward manor, the difference between the total number of foragers pre- and post-challenge was calculated (sum after - sum before) for each colony. Since the data was normally distributed, ANOVA was used to determine significant differences (JMP v.9.0) within each year. Data were then combined for unchallenged and CB-challenged treatments across years and analyzed using two-way ANOVA with year and CB-treatment as main effects. For the 2008 data, differences in parasite load were also examined using a two-way ANOVA with resin-status and CB-treatment as main effects.

In all years, the sample size included colonies in which resin foragers were observed in both pre- and post-challenge periods. Colonies that had clinical symptoms of CB or other infection (e.g., Deformed Wing Virus) during the pre-challenge period were not included in the analysis. A total of 5 colonies were removed from analyses (4 in 2008, 2 from each treatment, and 1 AFB-challenged colony in 2009). Since observations equaled approximately 1.5 hours per colony, and thus approximately 100 hours of field observations over the course of this experiment, the few colonies with zero foragers at one time point were likely either a result of missed sampling or another issue. This resulted in the following sample sizes: 2008— n = 7 unchallenged and n = 10 CB-challenged colonies; 2009— n = 8 unchallenged n = 8 AFB-challenged, n = 8 CB-challenged and n = 9 *Metarhizium*-challenged colonies; 2010— n = 6 unchallenged and n = 7 CB-challenged colonies.
